# Hepatocyte Growth Factor Modulates Interleukin-6 Production in Bone Marrow Derived Macrophages: Implications for Inflammatory Mediated Diseases

**DOI:** 10.1371/journal.pone.0015384

**Published:** 2010-11-02

**Authors:** Gina M. Coudriet, Jing He, Massimo Trucco, Wendy M. Mars, Jon D. Piganelli

**Affiliations:** 1 Department of Pathology, University of Pittsburgh School of Medicine, Pittsburgh, Pennsylvania, United States of America; 2 Department of Pediatrics, Children's Hospital of Pittsburgh of UPMC, Pittsburgh, Pennsylvania, United States of America; Centre de Recherche Public de la Santé (CRP-Santé), Luxembourg

## Abstract

The generation of the pro-inflammatory cytokines IL-6, TNF-α, and IL-1β fuel the acute phase response (APR). To maintain body homeostasis, the increase of inflammatory proteins is resolved by acute phase proteins via presently unknown mechanisms. Hepatocyte growth factor (HGF) is transcribed in response to IL-6. Since IL-6 production promotes the generation of HGF and induces the APR, we posited that accumulating HGF might be a likely candidate for quelling excess inflammation under non-pathological conditions. We sought to assess the role of HGF and how it influences the regulation of inflammation utilizing a well-defined model of inflammatory activation, lipopolysaccharide (LPS)-stimulation of bone marrow derived macrophages (BMM). BMM were isolated from C57BL6 mice and were stimulated with LPS in the presence or absence of HGF. When HGF was present, there was a decrease in production of the pro-inflammatory cytokine IL-6, along with an increase in the anti-inflammatory cytokine IL-10. Altered cytokine production correlated with an increase in phosphorylated GSK3β, increased retention of the phosphorylated NFκB p65 subunit in the cytoplasm, and an enhanced interaction between CBP and phospho-CREB. These changes were a direct result of signaling through the HGF receptor, MET, as effects were reversed in the presence of a selective inhibitor of MET (SU11274) or when using BMM from macrophage-specific conditional MET knockout mice. Combined, these data provide compelling evidence that under normal circumstances, HGF acts to suppress the inflammatory response.

## Introduction

As a first line of defense in response to infection, tissue injury and stress, macrophages generate the NFκB-dependent pro-inflammatory cytokines, TNF-α, IL-1β and IL-6[1]. The expression of these pro-inflammatory cytokines serves to facilitate the expeditious infiltration of immune cells by rapidly leading to an increase in blood flow and permeability into capillaries. The immune response is tightly regulated and dependent on signaling through ligands binding to Toll-like receptors (TLRs) on the surface of macrophages[2], [3]. Receptor/ligand interaction initiates a signaling cascade that involves the multifaceted enzyme, GSK3β that can then further modulate NFκB activity to transition between the generation of pro- and anti-inflammatory signals[4], [5], [6], [7]. Although necessary and beneficial during infection and tissue injury, the pro-inflammatory cytokine response must be resolved in order to reset the homeostatic threshold and subsequently repair affected tissues in the absence of excess inflammatory mediators[8], [9], [10].

IL-6 induction serves a dual role in the transition between propagation of the inflammatory response and initiation of the APR[9]. The APR serves to reset homeostasis after the ensuing inflammation by mediating the production of acute phase proteins from hepatocytes[8], [11], [12]. These proteins include plasminogen activator inhibitor type 1 (PAI-1) and urokinase plasminogen activator (uPA), both of which are involved in the regulation of hepatocyte growth factor (HGF) activity[12]. IL-6 also promotes HGF transcription and generation of the latent protein[13], [14], [15]. Since IL-6 production both promotes the increased generation of HGF and induces the APR, we hypothesized that accumulating HGF may act to resolve inflammation after stress.

To investigate this hypothesis we sought to assess the role of HGF and its cognate receptor, MET, with regard to innate immune activation of LPS-stimulated BMM. Our results demonstrate that in the presence of HGF there is a significant decrease in the secreted levels of IL-6, suggesting that HGF suppresses inflammation after injury. The suppression of IL-6 is achieved through HGF-dependent inactivation of GSK3β, a powerful governor of inflammatory signaling. This inactivation of GSK3β enhances the anti-inflammatory pathway by promoting the interaction of phospho-CREB with CBP and, occurs in concert with an overall decrease of phospho-p65 (Ser 276) and elevated levels of the anti-inflammatory cytokine IL-10[16], [17], [18], [19], [20]. Hence, our results indicate that HGF is a potent anti-inflammatory agent.

## Results

### HGF suppresses inflammation in bone marrow-derived macrophages

**Figure 1:**
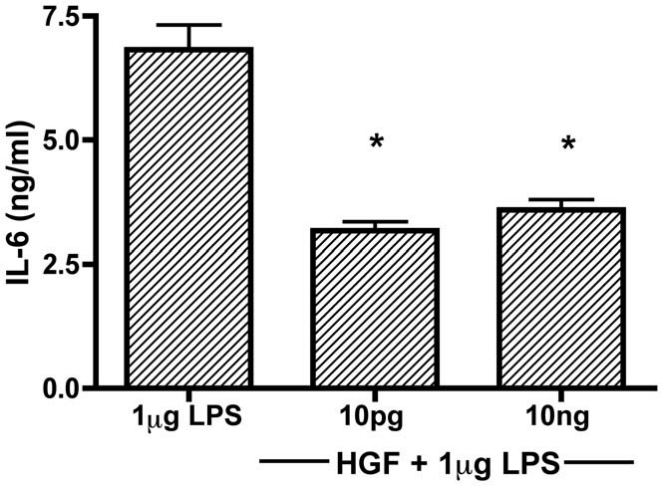
HGF modulates IL-6 production in LPS stimulated macrophages. BMM derived from C57BL6 mice were pretreated with or without 10 pg and 10 ng HGF for 24 hours and stimulated with 1 µg/ml LPS. Cell culture media was collected (24 h) and IL-6 levels were measured by ELISA. Results are representative of the mean (± SEM) of three independent experiment done in triplicate, * indicates <0.001.

In order to determine that HGF plays a role in regulating the APR to suppress inflammation, we utilized a well-defined *in vitro* model of acute inflammation: LPS stimulation of BMM. BMM were cultured with various physiological concentrations of HGF and then stimulated with LPS. Figure 1 demonstrates that 10 pg and 10 ng of HGF exhibit a significant suppression of IL-6 production in LPS-stimulated BMM after 24 hours.

### Pharmacological inhibition of HGF-MET signaling abolished HGF's suppressive effects in BMM

**Figure 2:**
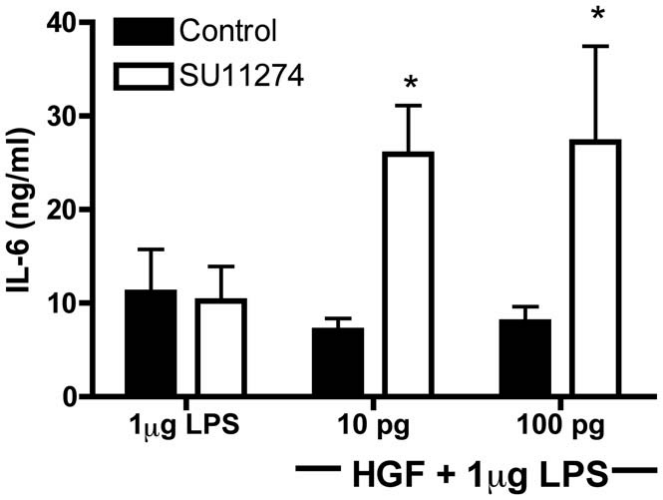
A MET kinase inhibitor abrogates HGF suppression of IL-6. Using an optimal dose of the MET inhibitor SU11274 (1 µg), BMM were pretreated for 2 hours before an overnight incubation with 10 and 100 pg HGF and 24 hour stimulation with LPS. Results are representative of the mean (± SEM) of three independent experiments done in triplicate. *, p = 0.02 vs. the respective control group.

To further confirm that the inhibition of IL-6 production was a result of HGF signaling, we repeated the *in vitro* model of acute inflammation, this time in the presence of SU11274, a specific MET inhibitor. An optimal concentration of 1 µM was chosen for the inhibition of signaling in BMM. SU11274[21], [22] was added to BMM cultures 2 hr prior to the addition of HGF and then cultures were stimulated with LPS. The results demonstrate that incubation with the MET inhibitor abolished the inhibitory effect induced by HGF on IL-6 production in BMM stimulated with LPS (Figure 2).

### Conditional deletion of MET-receptor on BMM confirms pharmacological data demonstrating HGF's suppressive effects in BMM

**Figure 3:**
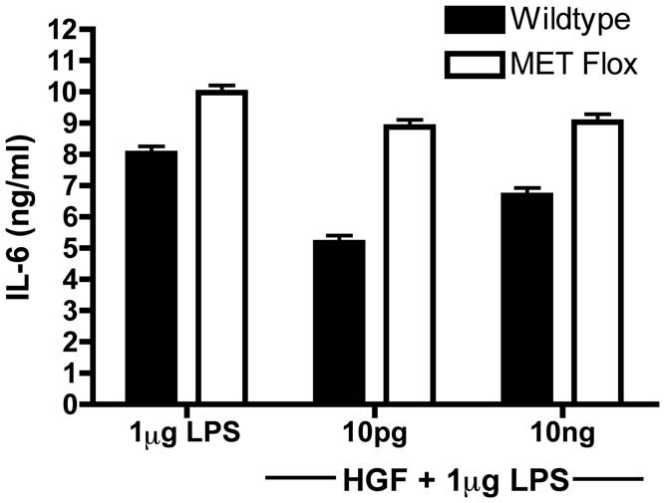
Deletion of the HGF receptor MET demonstrates a reversal in the effects of HGF on LPS stimulated BMM. BMM derived from either macrophage specific MET conditional knockout mice (MET^fl/fl^:cre^lysZ+/−^) or their wild type littermate controls (MET^fl/fl^:cre^lysZ−/−^) were pretreated with or without 1, 10 and 100 pg HGF for 24 hours and stimulated with 1 µg/ml LPS. Cell culture media was collected (24 h) and IL-6 levels were measured by ELISA. Results are representative of two independent experiments done in triplicate.

To rule out any offsite pharmacological effects by the MET inhibitor as the cause for the IL-6 inhibition and to further study the important role HGF plays in tempering the acute inflammatory response, conditional MET flox mice specific for the macrophage lineage were generated. Figure 3 demonstrates that BMM isolated from MET conditional knockout mice fail to suppress IL-6 production in response to LPS as compared to their wild type littermate controls. Use of the knockout animals confirmed the results demonstrated with the pharmacological MET inhibitor whereby the suppressive effect of HGF on IL-6 production was significantly reduced as compared with cultures not treated with the inhibitor (Figure 2). Taken together, these results clearly illustrate the interaction of HGF and MET suppresses IL-6, further supporting the important role HGF plays in tempering the acute inflammatory response.

### HGF suppresses inflammation through GSK3β

**Figure 4:**
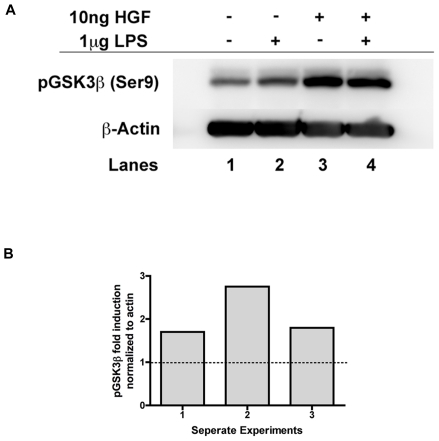
Treatment with HGF leads to increased GSK3β phosphorylation. (A) Cytoplasmic lysates prepared from BMM were incubated with 10 ng HGF for 24 hours prior to stimulation with LPS (1 µg/ml) for 15 minutes. The lysates were separated by SDS-PAGE and probed with a phospho-specific GSK3β antibody before re-probing for β-actin. (B) Densitometric analysis for phospho-GSK3β fold induction normalized to β-actin is shown for 3 separate experiments. Note that all experiments show induction greater than 1 when HGF is present.

To further understand the mechanism in which HGF suppresses the inflammatory response, we looked downstream of HGF-MET signaling at potential regulatory targets. One target, GSK3β, is known to regulate inflammation through activation of NFκB, resulting in pro-inflammatory cytokine production[5], [7], [23], [24], [25], [26]. When GSK3β is in its inactive state (phosphorylated), its influence over NFκB activation is limited and therefore pro-inflammatory cytokine production is quantitatively lessened[5]. GSK3β is known to be a downstream target of HGF[27], [28], [29]. We found that protein lysates prepared from BMM isolated from C57BL6 mice cultured with 10 ng HGF demonstrated an increased in phosphorylated, or inactive GSK3β (Figure 4), supporting the idea that HGF-MET interactions lead to inactivation of GSK3β.

### HGF signaling leads to the interaction of CBP with phospho-CREB by GSK3β

**Figure 5:**
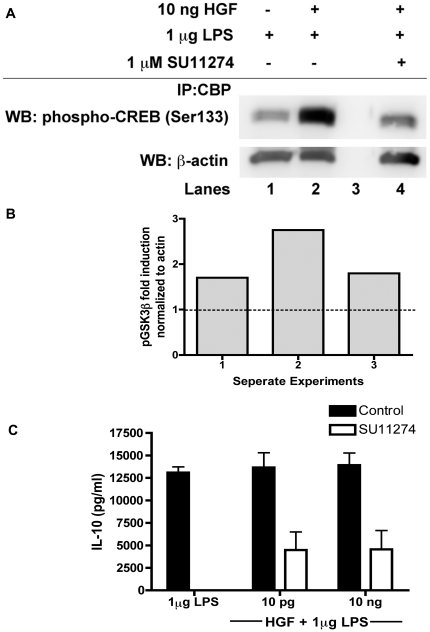
HGF promotes the interaction of phosphorylated CREB with CBP along with an increased production of IL-10. (A) Whole cell lysates prepared from BMM were incubated overnight with HGF (10 ng) prior to stimulation LPS (1 µg/ml) for 15 minutes, then subjected to immunoprecipitation with a CBP antibody. The lysates were separated by SDS-PAGE and probed with a phospho-specific CREB antibody before re-probing for β-actin. (B) Densitometric analysis for phospho-CREB fold induction normalized to β-actin. Is shown for 3 separate experiments. Note that all experiments show induction greater than 1 when HGF is present. (C) BMM were pretreated with or without 1 µM of the MET kinase inhibitor (SU11274) for 2 h prior to incubation with HGF (1, 10 and 100 pg) for 24 hours followed by stimulation with 1 µg/ml LPS. Cell culture media was collected (24 h) and IL-10 levels were measured by ELISA. Results are representative of two independent experiments done in triplicate.

To further determine the downstream signaling that results from HGF's regulation of GSK3β, we investigated the interaction of NFκB with the co-activator protein (CBP). Interactions between NFκB and CBP are facilitated by activated GSK3β when there is promotion of pro-inflammatory cytokine production[7], [24]. Our data, however, shows that the inhibition of GSK3β following HGF treatment is associated with an increased interaction between CBP and phospho-CREB. Stimulation of BMM with LPS in the presence of HGF led to an increase of phosphorylated (inactive) GSK3β (Ser 9) (Figure 4), which correlated with an increase in CBP-phospho-CREB (Ser 133) interaction (Figure 5A) followed by an increase in the levels of IL-10 production (Figure 5C).

### HGF inhibits the phosphorylation of Ser 276 on p65 of NFκB

**Figure 6:**
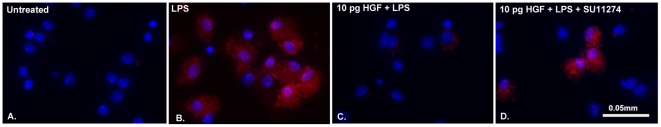
HGF prevents the nuclear translocation of phosphorylated p65. BMM were (A) untreated (B) stimulated with LPS (1 µg/ml) (C) treated with 10 pg HGF and stimulated with LPS (1 µg/ml) or (D) treated with the MET kinase inhibitor, SU11274, 10 pg HGF and stimulated with LPS (1 µg/ml). Cytospin preparations were then stained by immunoflourescence for phosphorylated p65 (Ser 276) and for nuclei with DAPI. The blue staining indicates nuclei, red staining indicates phosphorylated p65, and purple staining indicates colocalization of phospho-p65 within the nucleus.

The enhanced interaction between CBP and phospho-CREB during the HGF-induced anti-inflammatory response suggests CBP is being sequestered away from NFκB. Normally, the transcriptional activity of NFκB occurs through the phosphorylation of p65 at Serine 276 whereby a conformational change takes place that allows its nuclear association with CBP/p300[30]. This interaction allows for NFκB-dependent transcription of pro-inflammatory cytokines such as IL-6. Figure 6 demonstrates abundant phosphorylation of p65 at Ser 276 in both the nucleus and cytoplasm upon stimulation with LPS alone (Figure 6B). However, with the addition of HGF (10 pg) the overall phosphorylation of p65 is reduced with nuclear localization essentially absent (Figure 6C). Again, the effects of HGF are abrogated when SU11274 is added to the cultures, indicating the suppression is mediated via the HGF receptor, MET (Figure 6D).

## Discussion

IL-6, a key pro-inflammatory cytokine, is upregulated “as a defense mechanism” in order to promote the APR and to initiate homeostasis as quickly as possible after an acute injury. In cases of chronic stress; however, IL-6 changes its role by modulating the leukocytic repertoire resulting in a chronic inflammatory state[9], [11]. Although the presence of IL-6 is imperative during early injury and acute inflammation, contraction of the inflammatory process must occur in order for the system to regain normal homeostasis and to initiate repair. As a result, IL-6 is also posited to possess anti-inflammatory properties. Evidence to support this latter hypothesis is shown by the enhanced inflammatory responses induced following endotoxin exposure to IL-6^−/−^ mice[9], although, the authors never suggest a biochemical mechanism to explain why this unanticipated phenomenon occurs. HGF, a cytokine widely known for perpetuating liver regeneration, has also been described as having anti-inflammatory properties in cases of persistent inflammation[27], [28], [29], [31], [32], [33]. Importantly, the expression of HGF is induced by IL-6 while its regulation is controlled via acute phase proteins (urokinase and PAI-1) that are also induced following IL-6 stimulation. Hence, we posited a feedback loop wherein the pro-inflammatory properties ascribed to IL-6 are exhibited through induction of the APR and the anti-inflammatory properties are mediated via HGF that is produced in response to IL-6 stimulation.

Using LPS stimulated primary BMM cell cultures as a source of IL-6, we demonstrate that addition of HGF is in fact anti-inflammatory (Figure 1). Furthermore, we are able to confirm that the HGF-MET interaction propagates the suppression of cytokine production by using the pharmacological inhibitor of MET, SU11274 (Figure 2). Other studies have focused on using inhibitors of the PI3K and Akt signaling cascade[4], [7], that are down-stream of HGF-MET signaling; however, by using a direct MET kinase inhibitor as well as the macrophage specific MET floxed mice (Figure 3), we demonstrate that HGF-MET signaling is capable of suppressing inflammation.

Traditionally, GSK3β is known for regulating glycogen synthase and the storage of glycogen into peripheral sites[34], [35], but recent evidence suggests this kinase may also function as a key player in modulating inflammation[7], [17]. It is well known that the activation of NFκB through TLR signaling leads to the transcription of pro-inflammatory cytokines; however, recently data was published indicating that at a more general level, cytokine production is regulated through GSK3β, which in turn regulates NFκB activity[4], [7]. Hence, GSK3β appears to be a pivotal kinase that serves as a nodal point for both the generation and resolution of the inflammatory response[17], [27], [28]. Our data demonstrates that treatment BMM with HGF leads to increased phosphorylation and inactivation of GSK3β (Ser 9) (Figure 4) and that this response is sustained, even in the presence of LPS.

Pharmacological inhibitors of PI3K, Akt, and GSK3β induce inactivation of GSK3β. Inactive GSK3β can then promote the association of phospho-CREB (Ser 133) with CBP and sequester the CBP away from NFκB p65 (Ser 276). These signaling changes are associated with a switch from a pro- to anti-inflammatory pathway with a resultant increase in IL-10 production[7]. Our data shows that using HGF in place of those inhibitors gives similar results. BMM treated with both LPS and HGF demonstrated an increased CBP-phospho-CREB interaction, which was reduced in the presence of the MET kinase inhibitor (Figure 5). Furthermore, there was an increase in the production of IL-10 (Figure 5C) and, a reduction in the nuclear translocation phosphorylated p65. Combined, the data suggest that during an inflammatory response, active HGF may be key in switching the cellular response from a pro- to an anti-inflammatory pathway.

**Figure 7:**
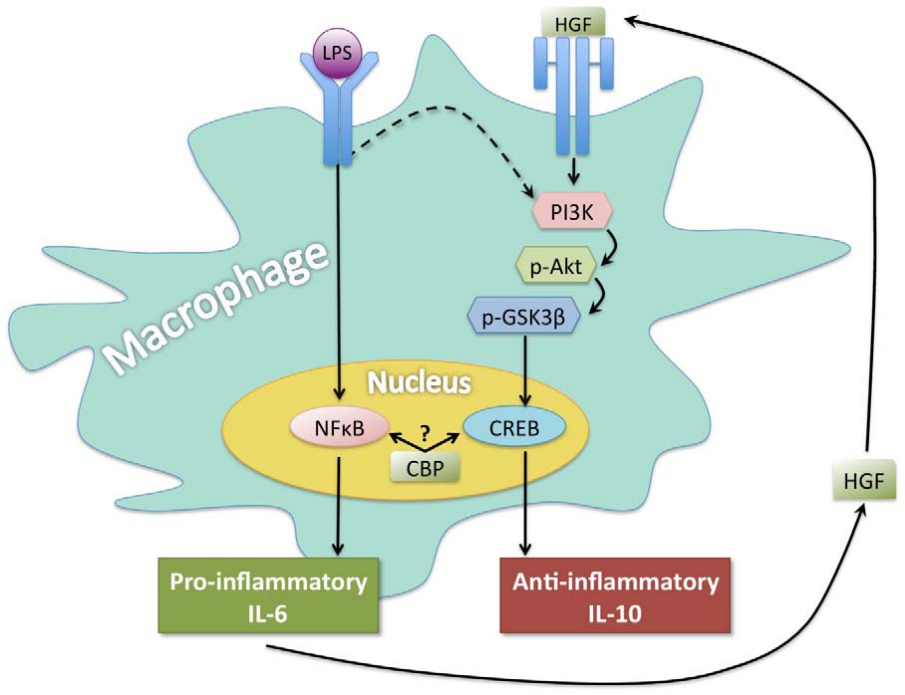
Proposed mechanism of HGF-mediated suppression. The canonical signaling pathway of LPS-TLR engagement leads to NFκB dependent pro-inflammatory cytokine production through the interaction of CBP with NFκB. However, TLR signaling has also been shown to weakly activate alternative signaling through PI3K, resulting in phosphorylation and inactivation of GSK3β, subsequent sequestration of CBP from NFκB to phospho-CREB, and resultant anti-inflammatory (IL-10) production[7]. Our results show that the presence of HGF enhances the IL-10 pathway. We postulate that in order to resolve inflammation, the generation of IL-6 by LPS-TLR signaling leads to the production of HGF, ultimately leading to the inhibition of inflammation. Hence, we propose HGF acts as an internal rheostat for resolving acute phase inflammatory responses.

In addition to the canonical pathway, TLR signaling has been shown to weakly activate PI3K. This then leads to anti-inflammatory events by altering the cytokine repertoire[7]. Hence, it has been postulated that PI3K is the point at which TLR signaling is differentiated from a pro-inflammatory to an anti-inflammatory condition. Our data indicates that in the absence of HGF, TLR signaling promotes the phosphorylation of NFκB along with its translocation to the nucleus and that this correlates with the production of the pro-inflammatory cytokine IL-6. In contrast, in the presence of HGF, GSK3β is phosphorylated (inactive) and TLR stimulation leads to production of the anti-inflammatory cytokine, IL-10. Notably, HGF signaling is well known to signal through the PI3K pathway[26], [27], [28], [36]. Hence, we propose that under normal circumstances, induction of IL-6 through pro-inflammatory stimuli leads to the eventual production of HGF[14], [15], [37]. HGF-MET interactions then ultimately result in phosphorylation of GSK3β and in the continued presence pro-inflammatory stimuli, facilitates an increased association of phospho-CREB with CBP. This then suppresses NFκB's transcriptional activity and results in the resolution of the inflammatory response (Figure 7). Our data describe the intimate interaction between IL-6 and HGF in regulation of inflammation. Hence we propose that HGF acts as an internal rheostat regulating the complex cascade of induction and resolution of inflammation (Figure 7).

## Materials and Methods

### Mouse strains

C57BL/6J and B6.129P2-Lyz2^tm1(cre)Ifo^/J mice were purchased from The Jackson Laboratory and the MET floxed mice were a gift from Dr. Snorri Thorgeirsson[38]. B6.129P2-Lyz2^tm1(cre)Ifo^/J were crossed with MET floxed mice to produce CRE^lysZ+^ MET^+/+^ animals. Male mice 6–8 week of age were used as a source of bone marrow-derived macrophages. All animals were housed under specific pathogen-free conditions in the Animal Facility at the University of Pittsburgh. This study was carried out in strict accordance with the recommendations in the Guide for the Care and Use of Laboratory Animals of the National Institutes of Health. The protocol was approved by the Institutional Animal Care and Use Committee of the University of Pittsburgh (Assurance Number A3187-01).

### Isolation of mouse bone marrow derived macrophages

Bone marrow derived macrophages (BMM) were cultured as previously described[39]. Femurs and tibias were dissected from sacrificed mice. Bone marrow cells were flushed from the bones using a 26 g needle until the bone is clear. The cells were then centrifuged and filtered through a cell strainer. Cells were cultured in 10% L929 conditioned media, with a media change every two days. Cultured cells were harvested and stained with the macrophage specific marker F4/80 to assess purity 7 days after isolation. Cells were plated on 24-well tissue culture plates at 1×10^6^ cells/well for supernatant analysis or on 100 cm tissue dishes at 2.5×10^7^ cells/dish for protein analysis.

### Preparation of samples for ELISA

BMM plated in 24-well plates were pretreated with recombinant mouse HGF (R&D Systems) for 24 h at 37°C prior to stimulation with 1 µg/ml lipopolysacharride (LPS) from *Escherichia coli* (055:B5) (Sigma Aldrich) for 24 h. In assays using the MET kinase inhibitor, SU11274 (Calbiochem), BMM were pretreated with 1 µM of the inhibitor for 2 h at 37°C followed by the 24 h HGF incubation and subsequent LPS stimulation. Cell culture supernatants were collected from triplicate wells, pooled and stored at −20°C for further analysis.

### Preparation of protein lysates

BMM were prepared as above and cultured in 100 mm dishes. At 15 minutes post-LPS stimulation, the BMM were washed with ice cold PBS and cells were harvested with gentle scraping and centrifugation. Cellular extracts were harvested using RIPA buffer supplemented with phosphatase and protease inhibitor cocktails (Roche) and 1 mM PMSF (Sigma-Aldrich). After the addition of 0.3 ml of lysis buffer, the macrophages were incubated on ice for 20 min, vortexed 3 times and centrifuged for 5 min at maximum speed at 4°C. The supernatant (whole cell lysate) was collected and the protein concentration of the lysates was determined by the bicinchonninic acid (BCA) protein assay according to the manufacturer's instruction (Pierce).

### Enzyme linked immunosorbent assay (ELISA)

Supernatants from BMM pretreated with HGF and/or SU11274 and stimulation with LPS were collected at 24 h. IL-6 and IL-10 cytokines secreted by BMM were measured by ELISA using purified capture and biotinylated detection antibody pairs (BD Pharmingen). The ELISA plates were read using a SpectraMax M2 plate reader, and the data was analyzed using SoftMax Pro software (Molecular Devices).

### Western immunoblotting

Protein lysates were assayed by Western blot as previously described, with minor exceptions[39]. Anti-phospho-GSK3β (Ser 9), anti-phospho-CREB (Ser 133) and anti-CREB used at 1∶1000 from Cell Signaling. Anti-GSK3β was used at 1∶1000 from Santa Cruz. Secondary antibodies were used from either Cell Signaling or Jackson ImmunoResearch. Blots were analyzed using the Fujifilm LAS-4000 imager and Multi Gauge software (Fujifilm Life Science).

### Immunoprecipitation

1 µg of CBP antibody (Santa Cruz) was added to 100 mg protein from whole cell lysates and incubated at 4°C overnight. The following day, Protein G-conjugated sepharose beads were added to the protein/antibody complex tube and incubated for 2 h. Immunoprecipitates were collected by centrifugation, washed twice with PBS, and then boiled in Laemmli sample buffer. Western blot was performed as described above.

### Cytospins

BMMs were washed with PBS and gently scraped from multi-well tissue culture dishes. Cells were collected into centrifuge tubes and spun at high speed for 10 min. Cell pellets were resuspended to 1×10^3^ cells per ml and cytospins were prepared with 100 µl of cells per slide. Slides were dried and stored at −80°C until staining.

### Phospho-NFκB p65 (Ser276) staining

Cytospun slides were fixed in 2% paraformaldehyde for 5 minutes and blocked with 20% non-immune normal goat serum for 1 hour at room temperature. After immune stained with primary antibody (Rabbit anti-Phospho-NFκBp65 (Ser276) 1∶100, (Cell Signaling Technology), slides were washed 5 times in PBS and incubated with secondary antibodies Cy3-conjugated goat anti-rabbit (Jackson ImmunoResearch). Nuclear staining (Hoechest staining, Molecular Probes) was performed. Images were viewed at 40x magnification and captured by using a Nikon confocal microscope (Nikon D-ECLIPSE C1, Japan).
